# Integrated Molecular Docking and Network-Based Analysis Reveals Multitarget Interaction Patterns of Nutraceutical Compounds in Intervertebral Disc Degeneration

**DOI:** 10.3390/biomedicines14050983

**Published:** 2026-04-24

**Authors:** Ersin Guner, Omer Faruk Yilmaz, Muharrem Furkan Yuzbasi, Mehmet Albayrak, Fatih Ugur, Ibrahim Yilmaz

**Affiliations:** 1 Department of Pharmacy, Konya Numune Hospital, Ministry of Health, Republic of Turkey, 42060 Konya, Turkey; 2 Clinics of Orthopaedic and Traumatology, Corlu State Hospital, Ministry of Health, Republic of Turkey, 59850 Tekirdag, Turkey; 3 Department of Neurosurgery, Kahramanmaras Sutcu Imam University School of Medicine, 46040 Kahramanmaras, Turkey; 4 Department of Medical Services and Techniques, Vocational School of Health Services, Istanbul Rumeli University, 34570 Istanbul, Turkey; 5 Department of Orthopedics and Traumatology, Kastamonu University School of Medicine, 37200 Kastamonu, Turkey; 6 Unit of Pharmacovigilance, Dr. Ismail Fehmi Cumalioglu City Hospital, Ministry of Health, Republic of Turkey, 59020 Tekirdag, Turkey

**Keywords:** intervertebral disc degeneration, molecular docking, network-based analysis, protein–protein interaction, hub gene analysis, extracellular matrix remodeling, inflammatory signaling, nutraceutical compounds

## Abstract

**Background**: Intervertebral disc degeneration (IVDD) is driven by the interplay between inflammatory signaling, extracellular matrix (ECM) degradation, and impaired cellular adaptation. Although several nutraceutical compounds have been reported to exert protective effects in IVDD-related models, their multitarget mechanisms within integrated molecular networks remain incompletely characterized. **Methods**: An in silico framework integrating molecular docking with network-based analyses was employed to evaluate resveratrol, quercetin, melatonin, curcumin, and baicalein against a predefined panel of IVDD-associated targets, within an exploratory in silico framework. Binding affinities and interaction profiles were assessed using molecular docking, followed by protein–protein interaction (PPI) network construction, Gene Ontology (GO) and Kyoto Encyclopedia of Genes and Genomes (KEGG) enrichment analyses, and hub gene identification. **Results**: Docking analyses revealed binding energies ranging from −4.59 to −13.25 kcal/mol, with curcumin and quercetin showing plausible docking poses across a subset of selected targets under the applied protocol. Network analysis showed a highly interconnected structure centered on key inflammatory regulators, including NFKB1, IL6, TNF, IL1B, STAT3, and NLRP3, together with ECM-associated components such as ACAN, COL2A1, SOX9, MMP13, and ADAMTS5. Enrichment analyses further suggested significant associations with inflammatory signaling pathways, cytokine regulation, and ECM organization. **Conclusions**: These findings are compatible with a distributed, multitarget interaction pattern of nutraceutical compounds within IVDD-associated molecular networks. By integrating molecular docking with network-based analyses, this study offers a system-level framework for interpreting previously reported effects within a disease-specific context. Docking-derived interaction patterns should be interpreted as qualitative and exploratory observations, as docking scores represent model-dependent estimates and do not establish comparable pharmacological effects across heterogeneous targets. The results should be considered hypothesis-generating and require experimental validation.

## 1. Introduction

Intervertebral disc (IVD) degeneration (IVDD) is a leading cause of chronic low back pain and disability worldwide, imposing a substantial socioeconomic burden [1,2]. At the cellular and molecular levels, IVDD is characterized by a progressive imbalance between anabolic and catabolic processes within the IVD, ultimately resulting in extracellular matrix (ECM) breakdown, loss of disc height, and impaired biomechanical function [3]. Nucleus pulposus (NP), annulus fibrosus (AF), and endplate cells are continuously exposed to mechanical loading, hypoxia, oxidative stress, inflammation, and aging, all of which converge on shared molecular pathways driving disc degeneration. Accordingly, elucidating the signaling networks regulating ECM homeostasis has become central to the development of effective disease-modifying strategies.

Recent investigations have identified numerous anabolic and catabolic mediators implicated in IVDD pathogenesis, including cartilage oligomatrix protein (COMP), chondroadherin (CHAD), matrix metalloproteinases (MMPs; MMP-7, MMP-13, and MMP-19), interleukin-1 beta (IL-1β), hypoxia-inducible factor-1 alpha (HIF-1α), type II collagen (COL2A1), SRY-related high-mobility group box 9 (SOX9), nuclear factor kappa B (NF-κB), bone morphogenetic protein-2 (BMP-2), signal transducer and activator of transcription 3 (STAT3), and the nucleotide-binding oligomerization domain leucine-rich repeat-containing protein family pyrin domain-containing 3 (NLRP3) inflammasome. Collectively, these molecules orchestrate the balance between ECM synthesis and degradation, thereby determining disc integrity or degeneration [4,5,6,7,8,9,10,11,12,13,14,15,16,17,18,19].

Anabolic signaling pathways, such as insulin-like growth factor-1 (IGF-1)-mediated phosphoinositide 3-kinase/protein kinase B (PI3K/Akt) [20], transforming growth factor-beta (TGF-β)/Smad [21], BMP-2 [22], and BMP-7, promote ECM synthesis by enhancing SOX9-driven expression of COL2A1 and aggrecan (ACAN), which are essential for NP hydration and load-bearing capacity. By contrast, catabolic signaling cascades, including mitogen-activated protein kinase (MAPK)/extracellular signal-regulated kinase (ERK)/c-Jun N-terminal kinase (JNK)/p38 [23], tumor necrosis factor-alpha (TNF-α) [24], IL-1β, interleukin-6 (IL-6) [25], NF-κB, and STAT3, upregulate MMP and a disintegrin and metalloproteinase with thrombospondin motifs (ADAMTS) expression, thereby accelerating ECM degradation and disc degeneration. The disruption of this finely regulated anabolic–catabolic balance is a hallmark of IVDD progression.

Accordingly, targeting ECM-degrading enzymes has emerged as a rational therapeutic strategy. In particular, ADAMTS-5 and NF-κB inhibitors [26,27] have attracted attention due to their ability to reduce ACAN degradation and preserve ECM integrity in AF and NP cells. Conversely, approaches aimed at enhancing ECM synthesis, including TGF-β agonists and Smad modulators, have been explored for their regenerative potential. However, increasing evidence suggests that inflammation-driven cell death mechanisms, including apoptosis, pyroptosis, necroptosis, and cellular senescence, also play critical roles in IVDD progression, often acting upstream of ECM degradation.

Among inflammatory regulators, the NLRP3 inflammasome has emerged as a key mediator linking oxidative stress, mitochondrial dysfunction, and inflammatory cell death in disc cells. Activation of NLRP3 promotes IL-1β release and pyroptosis, further amplifying catabolic signaling through NF-κB and MAPK pathways [28]. Consequently, therapeutic strategies capable of simultaneously suppressing inflammatory signaling, oxidative stress, and ECM degradation while preserving anabolic activity may be more effective than single-target approaches.

**Figure 1:**
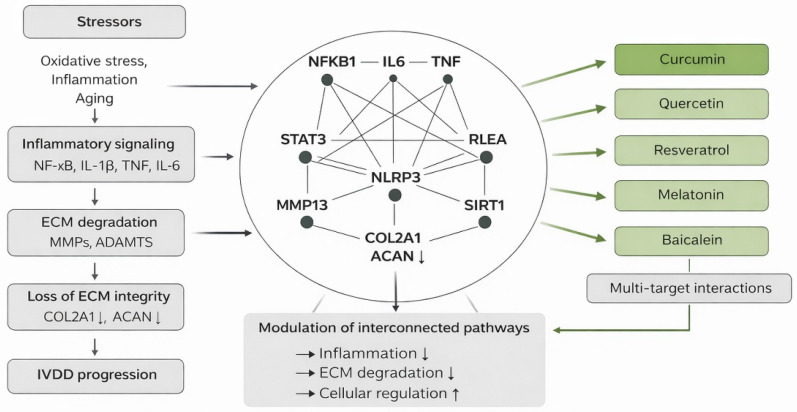
Schematic representation of IVDD-associated molecular network and multitarget interactions of nutraceutical compounds integrating inflammatory signaling, ECM remodeling, and regulatory pathways. Arrows indicate the direction of interactions and regulatory effects; upward arrows indicate activation or upregulation, whereas downward arrows indicate inhibition or downregulation.

In this context, nutraceutical compounds with pleiotropic biological activities have attracted growing interest as potential disease-modifying agents for IVDD. Resveratrol [29,30], quercetin [31], melatonin [32], curcumin [33], and baicalein [34,35,36] are among the most extensively studied nutraceuticals due to their well-documented antioxidant, anti-inflammatory, anti-senescent, and mitochondria-protective properties. These compounds have been shown to modulate key degenerative pathways, including NF-κB, MAPK, STAT3, and the NLRP3 inflammasome, while attenuating oxidative stress and reducing MMP/ADAMTS-mediated ECM degradation. Through these multitarget effects, nutraceuticals may help restore the anabolic–catabolic balance and support disc cell viability (Figure 1).

Despite increasing experimental evidence supporting their protective roles, the integrated molecular mechanisms and network-level interactions through which nutraceutical compounds exert their effects in IVDD remain incompletely characterized. In silico approaches, particularly when combining molecular docking with network-based analyses, provide a powerful and cost-effective strategy for predicting ligand-target interactions, identifying multitarget binding profiles, and elucidating the biological context of these interactions within complex signaling networks.

Therefore, the present study aimed to evaluate the in silico binding potential of resveratrol, quercetin, melatonin, curcumin, and baicalein against key IVDD-related molecular targets and to explore potential docking-derived interaction patterns within a predefined target set using protein–protein interaction (PPI) network analysis, functional enrichment, and hub gene identification.

## 2. Materials and Methods

### 2.1. Study Design

This study was designed as an in silico investigation integrating molecular docking with network-based analyses to characterize potential docking-derived interaction patterns of selected nutraceutical compounds in IVDD.

The nutraceutical compounds included in the present study were selected on a literature-guided basis. Resveratrol, quercetin, melatonin, curcumin, and baicalein were chosen because previous experimental and mechanistic studies have associated these compounds with anti-inflammatory, antioxidant, anti-catabolic, and cytoprotective effects relevant to IVDD-related pathways. Accordingly, compound selection was hypothesis-driven and exploratory rather than intended as an exhaustive screening of all possible nutraceutical candidates. The five compounds were evaluated against a predefined panel of IVDD-associated proteins. Docking-derived interaction profiles were subsequently interpreted in the context of PPI networks, functional enrichment analyses, and hub gene identification.

The target protein panel was defined a priori based on established biological relevance to IVDD, including inflammatory mediators, extracellular matrix-associated proteins, catabolic enzymes, and regulatory signaling molecules. This predefined selection was intended to reflect key pathobiological domains of disc degeneration rather than to perform an unbiased target discovery workflow.

This framework does not aim to construct a comprehensive drug–disease–target network; rather, it represents an integrative interpretation of docking-derived ligand–target interactions within a predefined IVDD-associated protein set and a STRING-derived PPI context.

#### 2.1.1. Molecular Docking Analysis

Molecular docking analyses were performed to evaluate potential interactions of resveratrol, quercetin, melatonin, curcumin, and baicalein with target proteins associated with IVDD. Docking simulations were carried out using AutoDock 4.2.6, (The Scripps Research Institute, La Jolla, CA, USA), and receptor and ligand structures were prepared using AutoDockTools (MGLTools, version 1.5.6; The Scripps Research Institute, La Jolla, CA, USA).

Ligand structures were prepared using AutoDockTools, with protonation states assigned under physiological pH assumptions. Dominant tautomeric forms were considered. Multiple ligand states were not explicitly enumerated; instead, a single representative conformation was used for docking following standard preparation procedures prior to PDBQT generation.

For each protein–ligand pair, docking was performed using the Lamarckian genetic algorithm with 10 runs, and the resulting poses were evaluated based on predicted binding energy (ΔG, kcal/mol), hydrogen bonding interactions, and RMSD.

Conformations with the lowest predicted binding energy and RMSD ≤ 2 Å were retained as representative poses for qualitative interaction analysis.

To assess the reliability of the docking protocol, a redocking procedure was performed for targets with available co-crystallized ligands. The native ligands were re-docked into their respective binding sites using the same docking parameters. The resulting poses were compared with the original crystallographic conformations based on RMSD. An RMSD threshold of ≤2 Å was adopted as a commonly accepted validation criterion for docking reproducibility. Redocking was applied as a target-level procedural validation step prior to subsequent docking analyses using the prepared receptor models, primarily to verify the consistency of binding site definition rather than to generate target-specific RMSD comparisons.

For metalloproteinase targets, catalytically relevant zinc ions present in the crystal structures were retained during receptor preparation. No specialized metal-aware docking protocol was applied; therefore, interactions involving metalloproteinases were interpreted cautiously.

Detailed docking parameters are provided in the Supplementary Materials. These supplementary data include grid box dimensions and centers, chain selection, docking parameters (GA runs, population size, and energy evaluations), ligand preparation procedures, and a description of the redocking validation procedure.

#### 2.1.2. Target Protein Selection

Three-dimensional (3D) structures of the target proteins were obtained from the PDB (https://www.rcsb.org). Catabolic and inflammatory targets included ADAMTS-5 (PDB ID: 6YJM) [37], MMP-7 (PDB ID: 7WXX) [38], MMP-13 (PDB ID: 2OW9) [39], MMP-19 (UniProt ID: Q99542), TNF-α (PDB ID: 2AZ5) [40], IL-6 (PDB ID: 1P9M) [41], IL-1β (PDB ID: 8C3U) [42], IKKβ (PDB ID: 4KIK) [43], NLRP3 (PDB ID: 9HG4) [44], and MD-2 (PDB ID: 2E59) [45].

For MMP-19, an AlphaFold-predicted structure was used due to the absence of an experimentally resolved PDB structure.

Anabolic and regulatory targets included HIF-1α (PDB ID: 4ZPR) [46], SIRT1 (PDB ID: 4I5I) [47], SOX9 (PDB ID: 4EUW) [48], and STAT3 (PDB ID: 6NJS) [49]. Structural ECM components included COL2A1 (PDB ID: 6JEC) [48] and ACAN (PDB ID: 9DFF) [50]. These components were included to explore structural interaction patterns rather than classical ligand–target pharmacological interactions.

#### 2.1.3. Ligand Preparation

Ligand structures were generated using ChemDraw Ultra 12.0 and optimized using ChemBio3D Ultra 13.0 with the MMFF94 force field. The optimized structures were converted to PDBQT format using Open Babel (version 3.1.1; Open Babel Development Team, USA).

#### 2.1.4. Molecular Docking Protocol

All water molecules were removed from the protein structures, and polar hydrogen atoms were added prior to docking. Catalytically essential metal ions (e.g., Zn^2+^ in metalloproteinases such as MMPs and ADAMTS-5) were retained during protein preparation to preserve the structural integrity of the active site. Protonation states of amino acid residues and ligands were assigned at physiological pH (7.4).

Grid boxes were defined to cover the active or functionally relevant regions of each protein. These regions were selected on the basis of co-crystallized ligand positions and/or literature-reported functional residues. The corresponding grid coordinates and chain selections are provided in the Supplementary Materials.

#### 2.1.5. Supplementary Data

Detailed protein-specific docking parameters, including grid box coordinates, chain selection, and structural preparation procedures, as well as comprehensive docking results (binding energies, RMSD values, and hydrogen bond interactions), are provided in the Supplementary Materials.

### 2.2. PPI Network Construction

The PPI network of the selected target proteins was constructed using the STRING database (version 12.0; https://string-db.org/, accessed on 21 March 2026) [51]. The analysis was restricted to Homo sapiens.

The minimum required interaction score was set to 0.7 (high confidence). Active interaction sources included experimental data, curated databases, co-expression, neighborhood, gene fusion, and co-occurrence. Disconnected nodes were excluded to improve network interpretability. Functionally associated proteins identified by the STRING algorithm were allowed to be included to improve network connectivity. The resulting network therefore reflects both the predefined target set and closely related interaction partners suggested by STRING. Network topology analysis was subsequently performed in Cytoscape (version 3.10.4; Cytoscape Consortium, San Diego, CA, USA). Hub genes identified by cytoHubba (e.g., TIMP1 and RELA) emerged from the interaction topology of the STRING-derived network.

### 2.3. Gene Ontology (GO) and Kyoto Encyclopedia of Genes and Genomes (KEGG) Pathway Enrichment Analysis

Functional enrichment analyses, including GO and KEGG pathway analyses, were performed using the STRING database. The statistical background was set to the whole genome. Enrichment significance was evaluated using FDR-adjusted *p*-values, and terms with FDR < 0.05 were considered statistically significant.

GO enrichment analysis was performed for the Biological Process (BP) category. KEGG pathway analysis was conducted to identify significantly associated signaling pathways.

The enrichment results were ranked based on FDR values, and the most relevant terms were selected for visualization. Functional enrichment visualization was performed using STRING built-in tools, with term grouping based on a similarity threshold of 0.8.

### 2.4. Network Visualization

The PPI network and enrichment results were visualized using STRING built-in visualization tools. Network topology parameters, including the number of nodes, number of edges, average node degree, and clustering coefficient, were automatically calculated. Enrichment plots were generated to display the most significant terms, where bubble size represents gene count and color intensity indicates statistical significance (FDR). Figures were exported in high-resolution formats for publication.

The PPI network was imported into Cytoscape [52] for visualization and topological analysis. Hub gene analysis was performed using the cytoHubba plugin (version 0.1; Cytoscape App Store, Boston, MA, USA) [53], and candidate hub genes were ranked according to the MCC algorithm.

In addition to hub gene identification, network topology parameters, including the number of nodes, number of edges, average node degree, and clustering coefficient, were evaluated. The interaction network was further analyzed to identify central inflammatory and ECM-related subnetworks.

In addition to the PPI-based representation, the analytical workflow conceptually reflects relationships between nutraceutical compounds, predefined IVDD-associated targets, and the disease context. Within this framework, docking-derived ligand–target associations may be interpreted as compound–target relationships, whereas the STRING-based PPI network represents disease-relevant target–target interactions. The integrative consideration of these components provides a system-level view linking compounds, targets, and IVDD-related biological processes. This representation was used to support exploratory interpretation rather than to construct an independent drug–disease–target network.

Accordingly, the present analysis should not be interpreted as an independent network pharmacology pipeline but as an integrative representation of predefined targets within a PPI context.

## 3. Results

### 3.1. Molecular Docking Results

Detailed docking results are provided in Supplementary Tables S1A–S1P (Supplementary Materials). Because the selected targets represent functionally diverse protein classes, including catalytic enzymes, signaling mediators, and structural extracellular matrix components, docking scores were interpreted cautiously. In particular, interactions with structural ECM proteins such as COL2A1 and ACAN were considered exploratory structural contact observations rather than classical pharmacological ligand–target interactions. Therefore, cross-target comparisons were not intended to imply comparable pharmacological potency but to provide a qualitative overview of potential interaction patterns within the predefined IVDD-related target panel.

**Figure 2:**
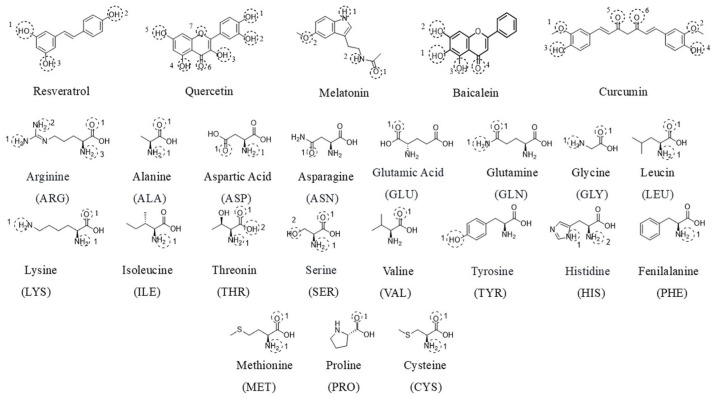
2D schematic representation of the selected nutraceutical compounds (resveratrol, quercetin, melatonin, curcumin, and baicalein) and representative amino acid residues involved in ligand–target interactions in IVDD-associated proteins.

A two-dimensional (2D) schematic diagram (Figure 2) presents the chemical structures of the selected nutraceutical compounds together with representative amino acid residues involved in ligand–target interactions.

Therefore, the observed docking patterns should be interpreted as qualitative interaction tendencies rather than as quantitative indicators of pharmacological potency.

### 3.2. Interaction with Catabolic Enzymes

**Figure 3:**
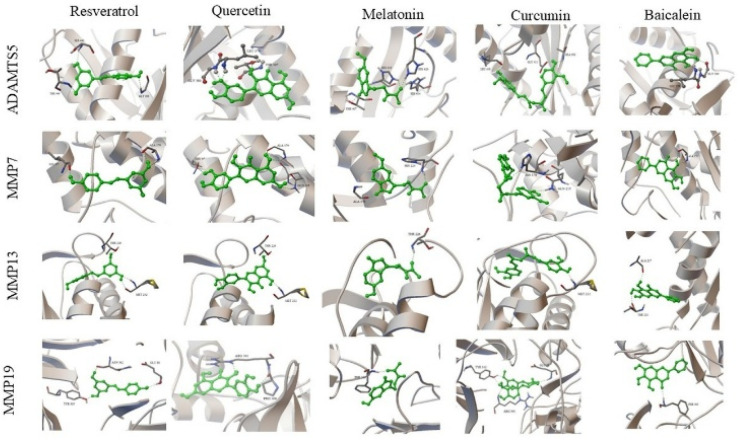
3D protein–ligand docking complexes formed by resveratrol, quercetin, melatonin, curcumin, and baicalein with the catabolic enzymes ADAMTS-5, MMP-7, MMP-13, and MMP-19 involved in IVDD. For each target protein, the ligands are superimposed at the same binding site. The positioning in the binding pockets, differences in orientation, and interactions with active site residues are shown. Green sticks represent ligands, while the protein structures are shown in ribbon/cartoon representation.

3D protein–ligand docking complexes illustrating the binding modes of resveratrol, quercetin, melatonin, curcumin, and baicalein with ADAMTS-5, MMP-7, MMP-13, and MMP-19 are presented in Figure 3. The ligands were predicted to occupy the active regions of the target proteins, forming binding poses consistent with surrounding amino acid residues.

Comparative visualization of the docking complexes showed differences in ligand orientation and interaction patterns within the binding pockets of the target proteins.

Analysis of docking scores indicated that quercetin exhibited binding energies of −12.32 kcal/mol for ADAMTS-5 and −13.25 kcal/mol for MMP-13, while curcumin exhibited binding energies of −10.52 kcal/mol for MMP-19 and −13.03 kcal/mol for MMP-13.

Multiple hydrogen bonding interactions were observed with residues including Met232, Thr226, Gly380, and Thr407.

### 3.3. Interaction with Pro-Inflammatory Cytokines

Docking interactions of the selected compounds with TNF-α, IL-1β, and IL-6 are presented below.

#### 3.3.1. TNF-α

Baicalein (−9.94 kcal/mol) and curcumin (−9.85 kcal/mol) exhibited the lowest binding energy values for TNF-α. These compounds interacted with residues including Tyr151 and Leu120 within the binding region of the protein.

#### 3.3.2. IL-1β

Quercetin (−10.86 kcal/mol) and baicalein (−10.10 kcal/mol) exhibited the lowest binding energy values for IL-1β. The ligands were located within the binding region and formed interactions with residues including Lys92, Lys93, and Met95.

#### 3.3.3. IL-6

**Figure 4:**
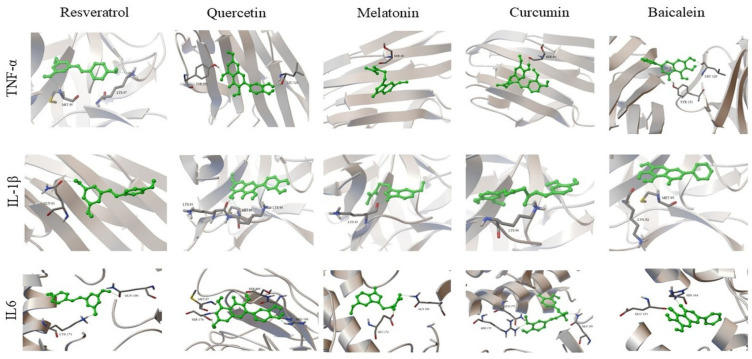
3D docking models of nutraceutical compounds with TNF-α, IL-1β, and IL-6, showing ligand binding conformations and interactions with surrounding amino acid residues. Green sticks represent ligands, while the protein structures are shown in ribbon/cartoon representation.

Curcumin exhibited the lowest binding energy value for IL-6 (−12.13 kcal/mol). Interactions were observed with residues including Arg179 and Gln190. 3D protein–ligand docking complexes of nutraceutical compounds with TNF-α, IL-1β, and IL-6 are presented in Figure 4.

### 3.4. Interactions with Inflammation and Cellular Stress Pathways

Docking interactions of the selected compounds with NLRP3, IKKβ, and MD-2 are presented below.

#### 3.4.1. NLRP3

Curcumin (−10.59 kcal/mol) and quercetin (−9.89 kcal/mol) exhibited the lowest binding energy values for NLRP3. Interactions were observed with residues including Ala99, Arg222, and Tyr503.

#### 3.4.2. IKKβ

Curcumin (−13.01 kcal/mol) and quercetin (−11.97 kcal/mol) exhibited the lowest binding energy values for IKKβ. Interactions with residues including Cys99 and Asp166 were observed.

#### 3.4.3. MD-2

**Figure 5:**
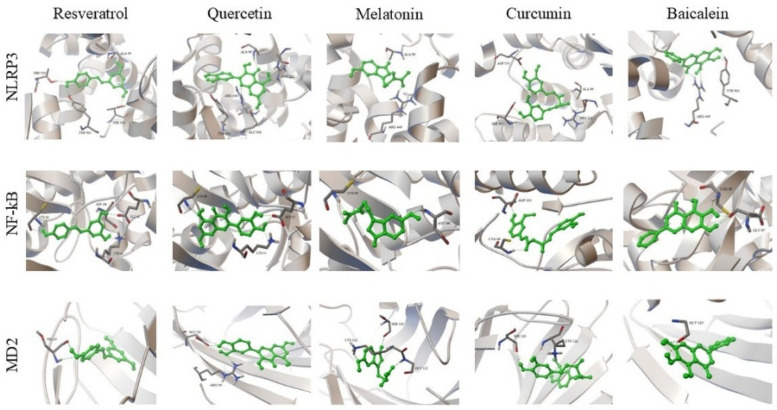
3D protein–ligand docking complexes of resveratrol, quercetin, melatonin, curcumin, and baicalein with NLRP3, IKKβ, and MD-2, showing ligand binding conformations and interactions with surrounding amino acid residues. Green sticks represent ligands, while the protein structures are shown in ribbon/cartoon representation.

Quercetin and baicalein exhibited binding energy values of −6.43 and −6.35 kcal/mol, respectively. Interactions within the binding region of MD-2 were observed. 3D docking models of nutraceutical compounds with NLRP3, IKKβ, and MD-2 are presented in Figure 5.

### 3.5. Interactions with Anabolic and Regenerative Signaling Targets

Docking interactions of the selected compounds with HIF-1α, SIRT1, STAT3, SOX9, COL2A1, and ACAN are presented below.

#### 3.5.1. HIF-1α

Curcumin (−12.17 kcal/mol) exhibited the lowest binding energy value for HIF-1α. Interactions were observed with residues in the Thr160–His166 region.

#### 3.5.2. SIRT1

Curcumin (−12.80 kcal/mol) and quercetin (−11.44 kcal/mol) exhibited the lowest binding energy values for SIRT1.

#### 3.5.3. STAT3

Quercetin (−8.69 kcal/mol) and curcumin (−8.23 kcal/mol) exhibited binding energy values for STAT3. Interactions were observed with residues including Gln644 and Tyr657.

#### 3.5.4. SOX9

Curcumin (−10.65 kcal/mol) and baicalein (−9.89 kcal/mol) exhibited the lowest binding energy values for SOX9. Interactions were observed within regions associated with the DNA-binding domain.

#### 3.5.5. COL2A1

Docking analysis of COL2A1 (PDB ID: 6JEC) showed binding energies ranging from −4.59 to −5.88 kcal/mol. Quercetin (−5.88 kcal/mol) and baicalein (−5.84 kcal/mol) exhibited the lowest binding energy values, while resveratrol (−4.59 kcal/mol) showed a higher binding energy value. RMSD values ranged from 0.06 to 1.22 Å.

#### 3.5.6. ACAN

Docking analysis of ACAN (PDB ID: 9DFF) revealed binding energies ranging from −7.90 to −9.89 kcal/mol. Quercetin (−9.89 kcal/mol) exhibited the lowest binding energy value. RMSD values ranged from 0.04 to 0.47 Å. Interactions were observed with residues including Ser125, Val154, Gln291, and Leu323.

**Figure 6:**
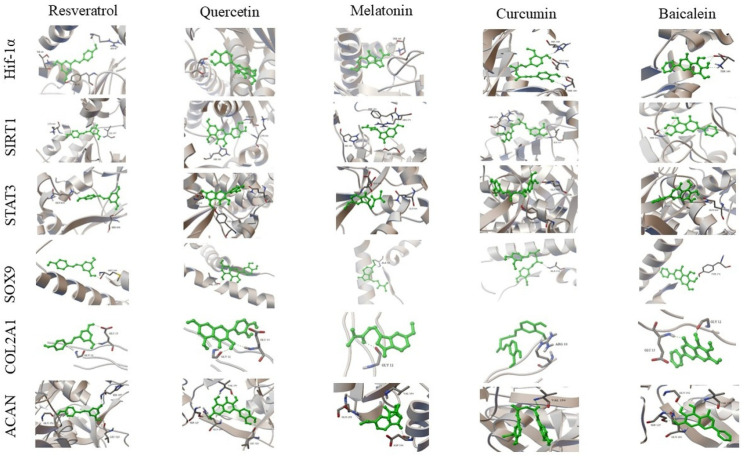
3D protein–ligand docking complexes of nutraceutical compounds with HIF-1α, SIRT1, STAT3, SOX9, COL2A1, and ACAN, showing ligand binding conformations and interactions with surrounding amino acid residues. Green sticks represent ligands, while the protein structures are shown in ribbon/cartoon representation.

3D protein–ligand docking complexes of nutraceutical compounds with HIF-1α, SIRT1, STAT3, SOX9, COL2A1, and ACAN are presented in Figure 6.

### 3.6. PPI Network Construction and Topological Analysis

**Figure 7:**
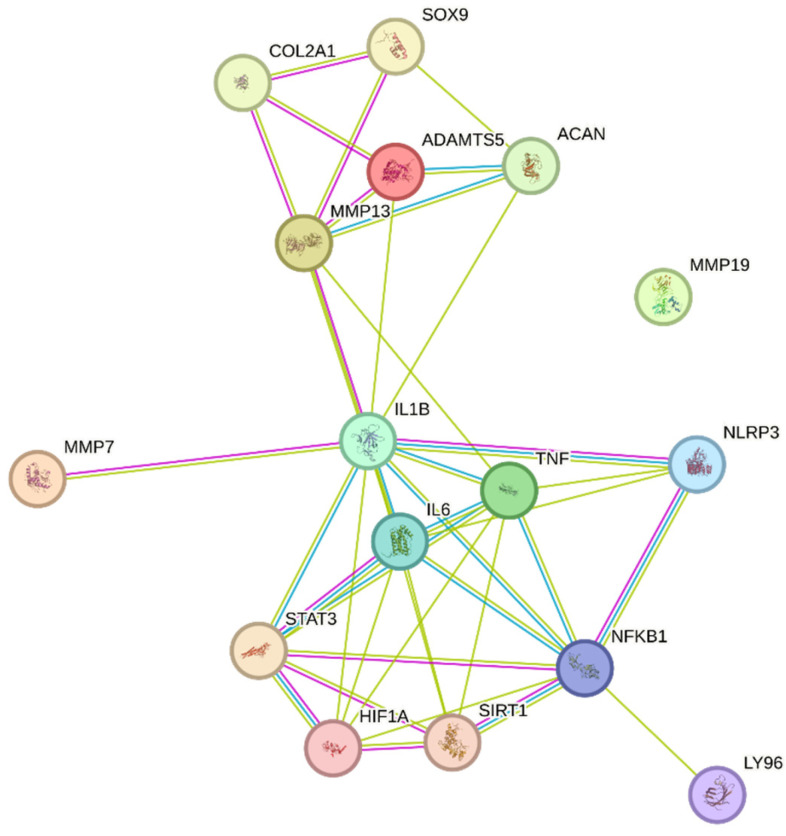
PPI network of the selected targets constructed using the STRING database. Nodes represent proteins, and edges indicate protein–protein associations. Edge colors represent different types of evidence for protein–protein associations as defined by the STRING database.

The PPI network constructed from the selected targets consisted of 21 nodes and 82 edges, with an average node degree of 7.81 and an average local clustering coefficient of 0.702. The number of observed interactions was significantly higher than expected (expected edges = 13), with a PPI enrichment *p*-value < 1.0 × 10^−16^ (Figure 7).

The increase in node number compared to the initial target set reflects the inclusion of functionally associated proteins identified by the STRING algorithm based on known and predicted interactions. These additional nodes were automatically incorporated by STRING based on known and predicted protein–protein associations and were not manually introduced. Topological analysis showed a densely interconnected core centered on NFKB1, IL1B, IL6, TNF, STAT3, and NLRP3. A distinct module composed of ACAN, COL2A1, SOX9, MMP13, and ADAMTS5 was also observed.

### 3.7. Gene Ontology (GO) and KEGG Pathway Enrichment Analysis

**Figure 8:**
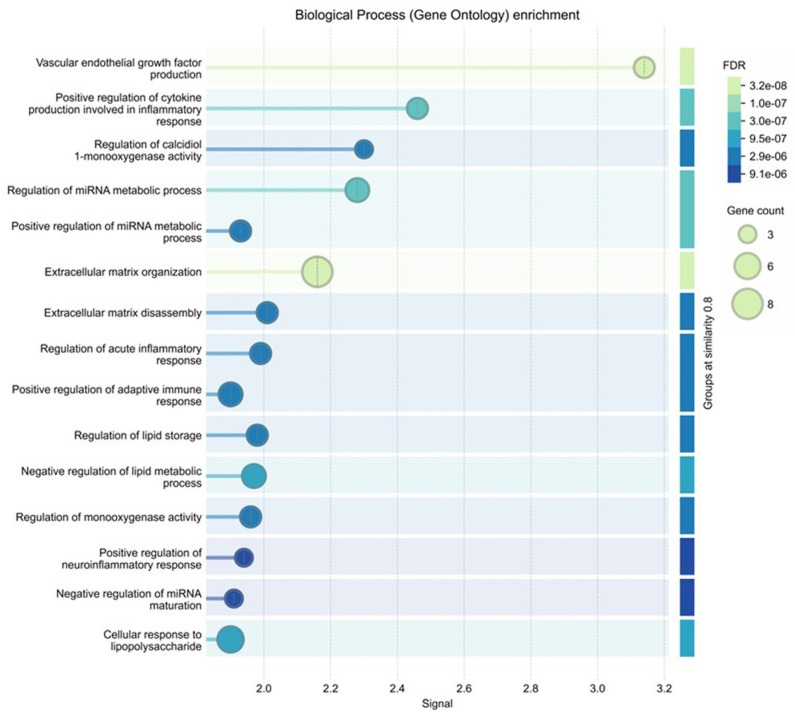
GO biological process enrichment analysis of the selected targets. The bubble plot shows enriched biological processes, where bubble size represents gene count and color indicates FDR.

GO biological process enrichment results are presented in Figure 8.

The most significantly enriched biological processes included response to oxygen-containing compound (FDR = 4.91 × 10^−10^), response to organic substance (FDR = 2.00 × 10^−9^), inflammatory response (FDR = 1.01 × 10^−8^), extracellular matrix organization (FDR = 2.56 × 10^−8^), and positive regulation of cytokine production (FDR = 5.27 × 10^−8^). Additional enriched processes included vascular endothelial growth factor production (FDR = 2.88 × 10^−7^), cartilage development (FDR = 3.37 × 10^−7^), and extracellular matrix disassembly (FDR = 1.24 × 10^−6^).

**Figure 9:**
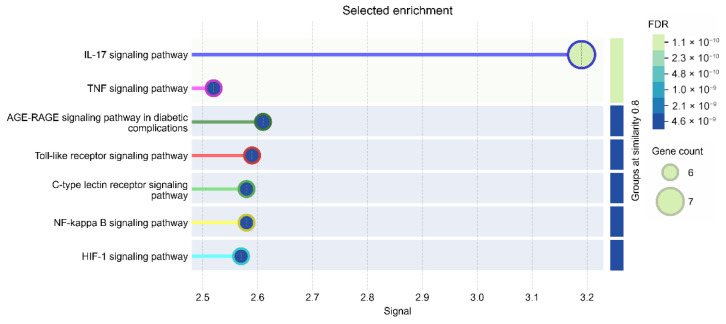
KEGG pathway enrichment analysis of the selected targets. The bubble plot shows enriched pathways, where bubble size corresponds to gene count and color represents FDR values.

KEGG pathway enrichment results are presented in Figure 9.

Significantly enriched pathways included IL-17 signaling pathway, Th17 cell differentiation, inflammatory bowel disease, NF-κB signaling pathway, TNF signaling pathway, Toll-like receptor signaling pathway, C-type lectin receptor signaling pathway, AGE–RAGE signaling pathway in diabetic complications, and HIF-1 signaling pathway.

The NOD-like receptor signaling pathway was also enriched (gene count = 6, FDR = 4.41 × 10^−7^) (Table 1).

### 3.8. Hub Gene Analysis

Hub gene analysis using the cytoHubba plugin in Cytoscape identified 10 hub genes based on the MCC algorithm (Table 2).

The MCC-based analysis ranked NFKB1, IL6, TNF, TIMP1, SIRT1, IL1B, STAT3, NLRP3, HIF1A, and RELA among the top-scoring nodes. Some hub genes (e.g., TIMP1 and RELA) were not part of the initial docking target list but emerged from the STRING-expanded network, reflecting their central positions within the broader interaction topology.

**Figure 10:**
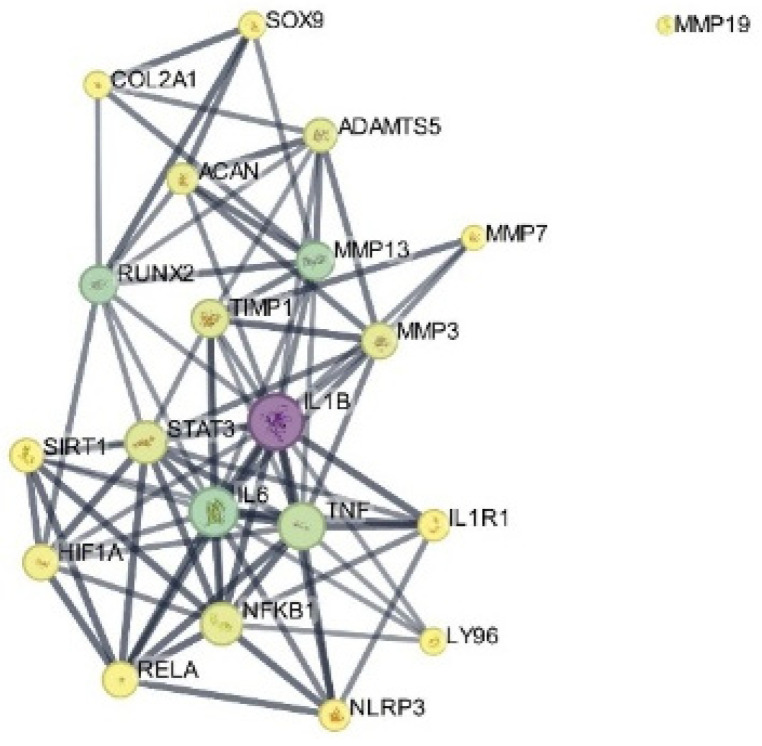
Cytoscape visualization of the hub-associated interaction subnetwork derived from the STRING PPI network. Hub genes identified using the cytoHubba plugin based on the MCC algorithm are highlighted. Node size represents degree centrality, and node color indicates betweenness centrality.

Visualization of the interaction network in Cytoscape is presented in Figure 10.

The interaction subnetwork included additional nodes such as MMP13, ADAMTS5, ACAN, COL2A1, and SOX9.

## 4. Discussion

IVDD is characterized by a progressive disruption of ECM homeostasis driven by the interplay between proteolytic activity, inflammatory signaling, and impaired cellular adaptation. MMPs and ADAMTS family members contribute to ECM degradation, whereas cytokine-driven pathways such as TNF-α, IL-1β, IL-6, and NF-κB sustain the degenerative cascade. In contrast, regulatory mechanisms involving SIRT1, SOX9, and HIF-1α have been associated with the maintenance of anabolic responses under hypoxic conditions [54].

Nutraceutical compounds including curcumin, resveratrol, quercetin, melatonin, and baicalein have been reported to exert anti-inflammatory and anti-catabolic effects in IVDD-related experimental models [55]. At the cellular level, these effects have been linked to reduced expression of inflammatory mediators [30], suppression of ECM-degrading enzymes [56], and attenuation of apoptosis [57]. These findings are further supported by studies describing the anti-catabolic effects of these compounds [58]. However, most of these observations have been interpreted within single-pathway frameworks rather than within an integrated systems context [59].

In the present study, molecular docking analyses suggested that curcumin and quercetin exhibited comparatively lower binding energy values across multiple targets, including ADAMTS-5 and MMP-13. At the same time, the binding energy values obtained in docking simulations should be interpreted cautiously, as they reflect structural compatibility rather than direct evidence of enzymatic inhibition.

A similar distribution of interactions was observed for inflammatory mediators, including TNF-α, IL-1β, IL-6, NLRP3, and IKKβ. Previous studies have shown that these compounds can modulate inflammatory signaling pathways in experimental settings [60]. Within this framework, the present findings should be considered as consistent with structural observations rather than direct functional validation.

The integration of docking results with PPI analysis provides a complementary perspective. The network structure suggested a densely interconnected core centered on NFKB1, IL1B, IL6, TNF, STAT3, and NLRP3, together with a module including ACAN, COL2A1, SOX9, MMP13, and ADAMTS5. The presence of inflammatory regulators and ECM-associated components within the same interaction network is compatible with a multi-layered organizational structure.

Within this context, the observed interaction profiles of curcumin and quercetin across multiple targets are compatible with a distributed interaction pattern rather than a single-target mechanism. This pattern is aligned with the concept of pleiotropic modulation, where compounds may interact with multiple nodes within interconnected biological pathways.

Functional enrichment analysis supports an interpretation compatible with this network-based interpretation. The identified targets were associated with pathways including NF-κB, TNF, IL-17, TLR, and NOD-like receptor signaling, as well as ECM organization and cytokine-related processes. These pathways have been consistently implicated in IVDD progression in previous studies [61].

Docking results obtained for structural ECM components, including COL2A1 and ACAN, should be interpreted with caution. These proteins do not represent classical pharmacological targets, and the observed interactions may be considered as potential ligand–protein contact patterns rather than evidence of direct functional modulation.

Taken together, the present study does not introduce novel molecular targets but provides a system-level interpretation of previously reported effects by integrating molecular docking with network-based analysis. Within this framework, curcumin and quercetin suggested comparatively favorable docking profiles among selected IVDD-related targets linked to inflammatory signaling and ECM remodeling.

Several limitations should be acknowledged. The selected target panel was defined based on known involvement in IVDD-related inflammatory and ECM-associated processes, rather than through an unbiased screening approach. In this context, the integration of molecular docking with PPI network analysis provides a complementary framework that is intended to contextualize the biological relevance of the selected targets at the systems level. All findings are based on in silico predictions and should therefore be considered hypothesis-generating. Molecular docking provides static estimates of binding affinity and does not account for protein flexibility, induced-fit effects, solvent interactions, or binding kinetics. In addition, molecular dynamics simulations were not performed in the present study. While such analyses may provide further insight into the stability of ligand–target interactions, the current in silico framework can be regarded as an exploratory, hypothesis-generating approach. Therefore, docking results should be interpreted cautiously, and additional computational and experimental validation, including molecular dynamics simulations, may be considered in future investigations. Furthermore, for metalloproteinase targets, the absence of specialized metal-aware docking treatment may represent an additional limitation. Consequently, docking results involving zinc-dependent enzymes should be considered qualitative and exploratory. The use of a predicted AlphaFold structure for MMP-19 introduces an additional level of structural uncertainty, and interactions observed for non-enzymatic ECM proteins should be interpreted cautiously. Furthermore, pharmacokinetic properties and long-term safety profiles of the investigated compounds were not evaluated. Accordingly, the enrichment results should be interpreted as reflecting the predefined IVDD-related target selection, and the present study provides an integrative, hypothesis-generating summary rather than an unbiased target discovery workflow. The present framework does not represent a full network pharmacology pipeline and should be interpreted within the boundaries of a predefined target-based analytical approach.

Future studies should focus on experimental validation using pathway-specific cellular assays and enzyme activity measurements. Moreover, the selected target panel included structurally and functionally heterogeneous proteins. Accordingly, docking-derived interaction patterns across different protein classes should be interpreted as qualitative and exploratory observations rather than as evidence of comparable pharmacological effects.

## 5. Conclusions

The present study integrates molecular docking with PPI and enrichment analyses to provide a network-based perspective on nutraceutical compounds in IVDD. The findings suggest that curcumin and quercetin were associated with plausible docking poses and comparatively favorable docking scores across a subset of selected IVDD-related targets under the applied protocol; these observations should be interpreted qualitatively and in an exploratory context, as docking scores represent model-dependent estimates and do not establish pharmacological potency or cross-target comparability. Rather than identifying novel molecular targets, this approach positions these compounds within an interconnected biological framework. The observed interaction patterns are compatible with multi-node structural engagement within predefined inflammatory, extracellular matrix–related, and cellular stress-associated pathways. All findings are based on computational predictions and should therefore be interpreted within a hypothesis-generating framework, and further experimental and mechanistic studies are required to validate the biological relevance of these observations.

## Figures and Tables

**Table 1:** Top enriched GO biological processes and KEGG pathways identified from STRING enrichment analysis.

Category	Term/Pathway	Gene Count	FDR
GO Biological Process	Response to oxygen-containing compound	16	4.91 × 10^−10^
GO Biological Process	Response to organic substance	18	2.00 × 10^−9^
GO Biological Process	Inflammatory response	11	1.01 × 10^−8^
GO Biological Process	Extracellular matrix organization	9	2.56 × 10^−8^
GO Biological Process	Positive regulation of cytokine production	10	5.27 × 10^−8^
GO Biological Process	Vascular endothelial growth factor production	4	2.88 × 10^−7^
GO Biological Process	Cartilage development	7	3.37 × 10^−7^
GO Biological Process	Extracellular matrix disassembly	5	1.24 × 10^−6^
KEGG Pathway	Pertussis	7	5.16 × 10^−10^
KEGG Pathway	IL-17 signaling pathway	7	1.11 × 10^−9^
KEGG Pathway	Th17 cell differentiation	7	1.30 × 10^−9^
KEGG Pathway	Inflammatory bowel disease	6	4.44 × 10^−9^
KEGG Pathway	NF-κB signaling pathway	6	4.64 × 10^−8^
KEGG Pathway	TNF signaling pathway	6	4.64 × 10^−8^
KEGG Pathway	Toll-like receptor signaling pathway	6	4.64 × 10^−8^
KEGG Pathway	C-type lectin receptor signaling pathway	6	4.64 × 10^−8^
KEGG Pathway	AGE–RAGE signaling pathway in diabetic complications	6	4.64 × 10^−8^
KEGG Pathway	HIF-1 signaling pathway	6	4.64 × 10^−8^
KEGG Pathway	NOD-like receptor signaling pathway	6	4.41 × 10^−7^

**Table 2:** Top 10 hub genes identified by cytoHubba using the MCC algorithm.

Rank	Gene Symbol	MCC Score
1	NFKB1	5280
2	IL6	5280
3	TNF	5280
4	TIMP1	5280
5	SIRT1	5280
6	IL1B	5160
7	STAT3	5160
8	NLRP3	5040
9	HIF1A	120
10	RELA	120

## Data Availability

The data supporting the findings of this study are available in the Supplementary Materials of this article. Additional data are available from the corresponding author on reasonable request.

## Supplementary Materials

The following supporting information can be downloaded at: https://www.mdpi.com/article/10.3390/biomedicines14050983/s1, Supplementary Methods; Tables S1A–S1P, including detailed descriptions as follows: Table S1A—ADAMTS-5 (PDB ID: 6YJM); Table S1B—MMP-7 (PDB ID: 7WXX); Table S1C—MMP-13 (PDB ID: 2OW9); Table S1D—MMP-19 (AlphaFold model; UniProt ID: Q99542); Table S1E—IL-6 (PDB ID: 1P9M); Table S1F—MD-2 (PDB ID: 2E59); Table S1G—TNF-α (PDB ID: 2AZ5); Table S1H—NLRP3 (PDB ID: 9HG4); Table S1I—IL-1β (PDB ID: 8C3U); Table S1J—NF-κB/IKKβ (PDB ID: 4KIK); Table S1K—HIF-1α (PDB ID: 4ZPR); Table S1L—SIRT1 (PDB ID: 4I5I); Table S1M—SOX9 (PDB ID: 4EUW); Table S1N—COL2A1 (PDB ID: 6JEC); Table S1O—ACAN (PDB ID: 9DFF); Table S1P—STAT3 (PDB ID: 6NJS).
