# Supplementary material for: Integrated Molecular Docking and Network-Based Analysis Reveals Multitarget Interaction Patterns of Nutraceutical Compounds in Intervertebral Disc Degeneration
Source: Biomedicines. 2026 Apr 24;14(5):983. doi: 10.3390/biomedicines14050983 (PMC13204500; doi:10.3390/biomedicines14050983)
Supplement: Supplementary file 1 [file biomedicines-14-00983-s001.zip › biomedicines-4242426-supplementary.pdf]

Supplementary Materials

Overview

This Supplementary File provides detailed molecular docking parameters and complete interaction datasets supporting the findings of the main manuscript. All docking analyses were performed under standardized conditions using identical algorithmic settings across all protein–ligand systems to improve procedural reproducibility. However, cross-target pharmacological comparability remains limited due to structural and functional heterogeneity of the selected targets.

Supplementary Methods

Molecular Docking Parameters

All molecular docking analyses were performed using identical algorithmic settings. Protein structures were obtained from the Protein Data Bank (PDB) or, when no experimentally determined structure was available, from an AlphaFold-predicted model.

Prior to docking, crystallographic water molecules and co-crystallized ligands were removed where applicable, and polar hydrogen atoms were added. Catalytically essential metal ions (e.g., Zn^{2+} in metalloproteinases) were retained to preserve the structural integrity of active sites.

Grid boxes were defined based on co-crystallized ligand positions and/or literature-reported active site residues to ensure biologically relevant docking regions.

Docking calculations were performed using the Lamarckian Genetic Algorithm implemented in AutoDock 4.2. For each protein–ligand pair, 10 independent GA runs were executed. The population size was set to 150 individuals, with a maximum number of 2,500,000 energy evaluations and 27,000 generations. Default values were used for mutation rate (0.02) and crossover rate (0.8). Other parameters were kept at AutoDock default settings.

Ligand Preparation

Ligand structures were energy-minimized using the MMFF94 force field. Protonation states were assigned at physiological pH (7.4), and structures were converted to PDBQT format prior to docking.

Docking Validation (Redocking Procedure)

To validate the reliability of the docking protocol, redocking was performed for protein structures with available co-crystallized ligands. Native ligands were re-docked into their original binding sites using identical docking parameters. The resulting poses were compared with crystallographic conformations based on root mean square deviation (RMSD).

An RMSD value ≤ 2 Å was considered indicative of acceptable reproducibility of the docking protocol. Redocking was used as a target-level procedural validation step before subsequent docking analyses were performed using the prepared receptor models. Target-specific RMSD values are available upon request, as redocking was used primarily as a procedural validation step rather than as a comparative metric across targets.

Protein-Specific Docking Parameters

HIF-1α (PDB ID: 4ZPR)

Chains A and B; crystallographic water molecules removed; polar hydrogen atoms added.

Grid center: x = −95, y = −34, z = 14 Å; grid size: 40 × 40 × 40 Å; spacing: 0.375 Å.

SOX9 (PDB ID: 4EUW)

DNA-bound monomeric chain; water molecules removed; polar hydrogen atoms added.

Grid center: x = −4, y = −19, z = 16 Å; grid size: 40 × 40 × 40 Å; spacing: 0.375 Å.

COL2A1 (PDB ID: 6JEC)

Trimeric structure composed of chains A, B, and C; docking performed to evaluate structural interaction potential.

Grid center: x = 0, y = 50, z = 3 Å; grid size: 40 × 40 × 40 Å; spacing: 0.375 Å.

ACAN (PDB ID: 9DFF)

Multiple chains (A–D); docking performed on chain A.

Grid center: x = 22.333, y = 0.361, z = 8.806 Å; grid size: 40 × 40 × 40 Å; spacing: 0.375 Å.

SIRT1 (PDB ID: 4I5I)

Two chains (A and B); docking performed on chain A.

Grid center: x = 46.22, y = −26.77, z = 17.97 Å; grid size: 40 × 40 × 40 Å; spacing: 0.375 Å.

STAT3 (PDB ID: 6NJS)

Single chain.

Grid center: x = 13, y = 54.5, z = 0.22 Å; grid size: 40 × 40 × 40 Å; spacing: 0.375 Å.

ADAMTS-5 (PDB ID: 6YJM)

Chains A and B; co-crystallized inhibitor removed prior to docking.

Grid center: x = 18.5, y = −5.944, z = 0.178 Å; grid size: 40 × 40 × 40 Å; spacing: 0.375 Å.

IL-6 (PDB ID: 1P9M)

Trimeric structure consisting of chains A–C; docking performed on chain B.

Grid center: x = −41.0, y = 177.0, z = 37.0 Å; grid size: 40 × 40 × 40 Å; spacing: 0.375 Å.

MD-2 (PDB ID: 2E59)

Single chain; co-crystallized lipid IVa removed prior to docking.

Grid center: x = −1, y = 12.61, z = 18.16 Å; grid size: 40 × 40 × 40 Å; spacing: 0.375 Å.

MMP-7 (PDB ID: 7WXX)

Single chain; inhibitor removed prior to docking.

Grid center: x = −34.75, y = −17.42, z = −8.50 Å; grid size: 40 × 40 × 40 Å; spacing: 0.375 Å.

MMP-13 (PDB ID: 2OW9)

Chains A and B; docking performed on chain A after inhibitor removal.

Grid center: x = 52.95, y = 10.92, z = 10.95 Å; grid size: 40 × 40 × 40 Å; spacing: 0.375 Å.

MMP-19 (AlphaFold model; UniProt ID: Q99542)

As no experimentally determined 3D structure of MMP-19 was available in the Protein Data Bank, an AlphaFold-predicted structure based on UniProt ID Q99542 was used.

Grid center: x = −7, y = 5, z = −4 Å; grid size: 40 × 40 × 40 Å; spacing: 0.375 Å.

NLRP3 (PDB ID: 9HG4)

Single chain.

Grid center: x = −17.05, y = −34.11, z = −5.08 Å; grid size: 40 × 40 × 40 Å; spacing: 0.375 Å.

TNF-α (PDB ID: 2AZ5)

Homotrimeric structure; docking performed on chain A.

Grid center: x = −18.61, y = 74.75, z = 33.22 Å; grid size: 40 × 40 × 40 Å; spacing: 0.375 Å.

IKKβ (PDB ID: 4KIK)

Dimeric structure; co-crystallized ligand removed prior to docking.

Grid center: x = 47.27, y = 30.41, z = −56.03 Å; grid size: 40 × 40 × 40 Å; spacing: 0.375 Å.

IL-1β (PDB ID: 8C3U)

Chains A and B; inhibitor removed prior to docking.

Grid center: x = −16.44, y = −4.361, z = −41.50 Å; grid size: 40 × 40 × 40 Å; spacing: 0.375 Å.

Supplementary Results

Docking Interaction Profiles

Supplementary Tables S1A–S1P present the complete molecular docking interaction profiles for each protein target, including binding energy (kcal/mol), RMSD values (Å), and hydrogen bond interactions.

TABLE S1A – ADAMTS-5 (PDB ID: 6YJM)

Compound	Binding Energy (kcal/mol)	RMSD (Å)	Hydrogen Bond Residues (Å)
Resveratrol	-9.59	0.32	SER440 (2.213), GLY380 (2.10), THR444 (2.11)
Quercetin	-12.32	0.11	LEU379 (1.80), GLY380 (1.65), THR407 (1.89)
Melatonin	-9.50	0.90	HIS410 (2.03), HIS414 (2.22), HIS420 (1.79), THR407 (1.89)
Curcumin	-11.79	1.06	ALA382 (1.17), GLU411 (2.04), LEU438 (2.74)
Baicalein	-11.06	0.06	LEU379 (1.89), GLY380 (2.14)

TABLE S1B – MMP-7 (PDB ID: 7WXX)

Compound	Binding Energy (kcal/mol)	RMSD (Å)	Hydrogen Bond Residues (Å)
Resveratrol	-7.24	1.27	ALA179 (2.01, 1.84), LEU97 (1.79)
Quercetin	-9.26	0.37	LEU97 (2.18), GLN215 (2.13, 2.11, 1.92)
Melatonin	-7.18	0.45	HIS224 (2.11), ALA179 (1.71)
Curcumin	-8.58	1.45	LYS122 (2.11, 2.02)
Baicalein	-7.85	0.07	ALA179 (1.64, 1.94)

TABLE S1C – MMP-13 (PDB ID: 2OW9)

Compound	Binding Energy (kcal/mol)	RMSD (Å)	Hydrogen Bond Residues (Å)
Resveratrol	-10.92	0.16	THR226 (2.10, 1.93), MET232 (2.02, 2.16)
Quercetin	-13.25	0.22	MET232 (1.87, 2.01)
Melatonin	-9.98	1.10	THR226 (1.89)
Curcumin	-13.03	0.47	MET232 (1.71)
Baicalein	-12.27	0.10	THR224 (2.14), ALA217 (2.01)

TABLE S1D – MMP-19 (AlphaFold model; UniProt ID: Q99542)

Compound	Binding Energy (kcal/mol)	RMSD (Å)	Hydrogen Bond Residues (Å)
Resveratrol	-8.40	0.27	GLU88 (2.15), TYR305 (2.02), ASN362 (2.14)
Quercetin	-9.75	0.40	ARG301 (2.07, 2.17), PRO300 (2.17)
Melatonin	-7.95	0.04	PHE363 (1.92)
Curcumin	-10.52	0.05	GLU88 (2.22), ARG301 (2.09), TYR342 (2.11, 2.15)
Baicalein	-9.88	0.08	PHE363 (2.06)

TABLE S1E – IL-6 (PDB ID: 1P9M)

Compound	Binding Energy (kcal/mol)	RMSD (Å)	Hydrogen Bond Residues (Å)
Resveratrol	-7.50	0.44	LYS171 (2.07), GLN190 (1.62, 2.14)
Quercetin	-10.05	0.09	ARG168 (2.01), MET67 (2.21), SER169 (2.03), SER176 (2.25)
Melatonin	-9.14	0.05	GLN190 (1.89), GLU172 (2.12)
Curcumin	-12.13	0.32	ARG179 (2.16), GLN190 (2.25), GLN175 (2.05)
Baicalein	-9.73	0.38	HIS164 (1.98), GLU51 (1.89)

TABLE S1F – MD-2 (PDB ID: 2E59)

Compound	Binding Energy (kcal/mol)	RMSD (Å)	Hydrogen Bond Residues (Å)
Resveratrol	-5.24	0.78	SER120 (2.19, 2.13)
Quercetin	-6.43	0.33	ARG90 (2.09), GLU92 (2.12, 1.91)
Melatonin	-5.42	1.74	LYS122 (2.11), GLY123 (1.75), SER120 (1.80)
Curcumin	-5.71	1.86	LYS122 (1.67), SER120 (2.07)
Baicalein	-6.35	0.27	GLY123 (2.13, 2.23)

TABLE S1G – TNF-α (PDB ID: 2AZ5)

Compound	Binding Energy (kcal/mol)	RMSD (Å)	Hydrogen Bond Residues (Å)
Resveratrol	-8.27	0.15	GLN61 (2.03)
Quercetin	-9.82	0.17	TYR151 (1.91), LEU120 (2.00, 2.14)
Melatonin	-8.13	0.15	SER60 (2.18)
Curcumin	-9.85	1.11	SER60 (2.22)
Baicalein	-9.94	0.04	LEU120 (1.89, 1.88), TYR151 (2.09)

TABLE S1H – NLRP3 (PDB ID: 9HG4)

Compound	Binding Energy (kcal/mol)	RMSD (Å)	Hydrogen Bond Residues (Å)
Resveratrol	-8.49	0.18	THR530 (2.14), TYR503 (2.00), ALA99 (2.09), THR310 (1.96)
Quercetin	-9.89	0.06	TYR503 (2.01), ALA99 (1.95), ARG222 (1.96, 2.08), ARG449 (1.86), GLU500 (2.20)
Melatonin	-8.03	1.04	ARG449 (2.13), ALA99 (1.99)
Curcumin	-10.59	1.66	ALA99 (1.80), ARG222 (2.10), SER497 (1.82, 1.86), ASP533 (1.84)
Baicalein	-8.90	0.13	ARG449 (2.12, 2.13), TYR503 (1.84, 1.98)

TABLE S1I – IL-1β (PDB ID: 8C3U)

Compound	Binding Energy (kcal/mol)	RMSD (Å)	Hydrogen Bond Residues (Å)
Resveratrol	-8.31	0.35	MET95 (2.14), LYS97 (2.20)
Quercetin	-10.86	0.06	LYS94 (1.87), LYS93 (2.24), MET95 (2.19)
Melatonin	-8.03	0.69	LYS93 (2.22)
Curcumin	-9.86	1.60	LYS94 (2.19)
Baicalein	-10.10	0.12	MET95 (1.79), LYS92 (2.10)

TABLE S1J – NF-κB / IKKβ (PDB ID: 4KIK)

Compound	Binding Energy (kcal/mol)	RMSD (Å)	Hydrogen Bond Residues (Å)
Resveratrol	-9.89	0.08	CYS99 (1.90, 2.23), LYS44 (2.10), ASP166 (2.15), GLU61 (2.08)
Quercetin	-11.97	0.14	LYS44 (1.96), CYS99 (1.65), GLU61 (2.21, 2.09)
Melatonin	-8.81	0.74	CYS99 (1.86), ASP166 (2.10)
Curcumin	-13.01	0.34	CYS99 (1.93), ASP103 (1.84)
Baicalein	-10.18	0.29	CYS99 (1.69), GLU97 (1.78, 2.17)

TABLE S1K – HIF-1α (PDB ID: 4ZPR)

Compound	Binding Energy (kcal/mol)	RMSD (Å)	Hydrogen Bond Residues (Å)
Resveratrol	-8.94	0.43	THR151 (1.92), THR160 (1.68), TYR111 (2.03), ARG68 (2.04)
Quercetin	-10.89	1.51	THR160 (1.88, 1.75, 1.78)
Melatonin	-7.93	1.76	THR160 (2.14)
Curcumin	-12.17	0.50	THR160 (2.13, 1.83), GLU163 (1.81), HIS166 (2.04)
Baicalein	-9.33	0.13	THR160 (2.12, 2.00, 1.96)

TABLE S1L – SIRT1 (PDB ID: 4I5I)

Compound	Binding Energy (kcal/mol)	RMSD (Å)	Hydrogen Bond Residues (Å)
Resveratrol	-8.92	0.44	ILE347 (2.12), LYS444 (1.92)
Quercetin	-11.44	0.04	HIS363 (2.16), SER442 (2.05, 2.17), LYS444 (1.91)
Melatonin	-8.90	1.24	PHE273 (2.19), ARG274 (1.90), HIS363 (2.05), SER441 (2.14)
Curcumin	-12.80	1.42	ARG274 (1.87, 2.24, 1.93), ILE347 (1.93)
Baicalein	-10.48	0.13	SER442 (1.76, 1.90, 1.74)

TABLE S1M – SOX9 (PDB ID: 4EUW)

Compound	Binding Energy (kcal/mol)	RMSD (Å)	Hydrogen Bond Residues (Å)
Resveratrol	-9.15	0.56	MET109 (1.96)
Quercetin	-9.84	0.26	VAL155 (2.04)
Melatonin	-8.80	1.86	ALA111 (2.12)
Curcumin	-10.65	0.44	ALA111 (2.12)
Baicalein	-9.89	0.10	TYR174 (2.18)

TABLE S1N – COL2A1 (PDB ID: 6JEC)

Compound	Binding Energy (kcal/mol)	RMSD (Å)	Hydrogen Bond Residues (Å)
Resveratrol	-4.59	0.51	GLU13 (1.82, 2.16), GLY12 (1.94)
Quercetin	-5.88	0.16	GLU13 (2.07), GLY12 (2.13)
Melatonin	-5.49	1.22	GLY12 (1.89)
Curcumin	-5.84	1.03	ARG10 (1.91)
Baicalein	-5.84	0.06	GLU13 (1.97), GLY12 (1.98)

TABLE S1O – ACAN (PDB ID: 9DFF)

Compound	Binding Energy (kcal/mol)	RMSD (Å)	Hydrogen Bond Residues (Å)
Resveratrol	-7.91	0.47	HIS197 (2.02), GLY152 (1.99), LEU323 (2.06)
Quercetin	-9.89	0.28	SER125 (1.77), VAL154 (2.17, 1.92), GLN291 (1.78), LEU323 (1.67, 1.67)
Melatonin	-7.90	0.11	GLN291 (2.06), VAL154 (1.95), ASP194 (1.84)
Curcumin	-8.66	1.31	VAL154 (2.49)
Baicalein	-9.48	0.04	SER125 (2.03), GLN291 (1.76), VAL154 (1.98), GLY152 (2.22)

TABLE S1P – STAT3 (PDB ID: 6NJS)

Compound	Binding Energy (kcal/mol)	RMSD (Å)	Hydrogen Bond Residues (Å)
Resveratrol	-6.79	0.48	GLN644 (2.15), SER636 (1.97)
Quercetin	-8.69	0.03	SER636 (1.89), GLN644 (1.97, 2.03), TYR657 (2.00, 2.00)
Melatonin	-7.52	0.51	GLN644 (2.24), GLU638 (2.13)
Curcumin	-8.23	0.82	GLN644 (1.99), TYR657 (1.93)
Baicalein	-7.58	0.14	GLN644 (2.15, 2.17)

Supplementary Classification Note

The docking targets presented in Supplementary Tables S1A–S1P were grouped according to their functional roles in IVDD pathophysiology. Tables S1A–S1D represent catabolic enzymes involved in extracellular matrix degradation. Tables S1E–S1J include inflammatory mediators and inflammation-related signaling proteins. Tables S1K–S1M comprise regulatory and transcription-associated targets

involved in cellular stress responses and tissue homeostasis. Tables S1N–S1O correspond to structural extracellular matrix components. Table S1P represents STAT3, a signaling regulator that functionally bridges inflammatory and regulatory pathways in IVDD.

Supplementary Note

The docking results presented in this Supplementary File represent computational predictions of ligand–protein interactions and should be interpreted as structural indicators supporting biological plausibility rather than direct evidence of functional activity.

Hydrogen bond interactions were identified based on distance criteria (≤3.5 Å) using docking pose analysis. All interactions are reported at the atom level based on docking pose geometry.
